# Commentary on: The Combined Effect of Intravenous and Topical Tranexamic Acid in Liposuction: A Randomized Double-Blinded Controlled Trial

**DOI:** 10.1093/asjof/ojab003

**Published:** 2021-01-12

**Authors:** Rafael A Couto

Please watch a video commentary on “The Combined Effect of Intravenous and Topical Tranexamic Acid in Liposuction: A Randomized Double-Blinded Controlled Trial” ^1^ featuring Dr Rafael A. Couto, MD (Video).
